# B Cell Responses to the Placenta and Fetus

**DOI:** 10.1146/annurev-pathmechdis-111523-023459

**Published:** 2025-01-02

**Authors:** Gabrielle Rizzuto

**Affiliations:** 1 Immuno-Oncology, Human Oncology and Pathogenesis Program, Memorial Sloan Kettering Cancer Center, New York, NY, USA; 2 Department of Anatomic Pathology and Laboratory Medicine, Memorial Sloan Kettering Cancer Center, New York, NY, USA; 3 Immunology and Microbial Pathogenesis Program, Weill Cornell Medicine Graduate School of Medical Sciences, New York, NY, USA

**Keywords:** fetomaternal tolerance, B cells, antibodies, trophoblast, glycosylation, adverse pregnancy outcomes

## Abstract

Pregnancy has fascinated immunologists ever since Peter Medawar’s observation that reproduction runs contrary to the founding tenets of immunology. During healthy pregnancy, maternal B cells interact with antigens of the foreign conceptus (placenta and fetus) yet do not elicit rejection. Instead, robust and redundant fetomaternal tolerance pathways generally prevent maternal B cells and antibodies from harming the placenta and fetus. Fetomaternal tolerance is not absolute, and unfortunately there exist several pregnancy complications that arise from breaks therein. Here, important historic and recent developments in the field of fetomaternal tolerance pertaining to maternal B cells and antibodies are reviewed. General rules from which to conceptualize humoral tolerance to the placenta and fetus are proposed. Significant but underexplored ideas are highlighted and topics for future research are suggested, findings from which are predicted to provide insight into the fundamental nature of tolerance and bolster efforts to combat immune-mediated pregnancy complications.

There is clearly a well-organized means of rendering any physiological association of maternal and fetal cells during pregnancy innocuous to both individuals.

—Sir Frank MacFarlane Burnet, 1969 (1, p. 567)

## INTRODUCTION

**Figure 1:**
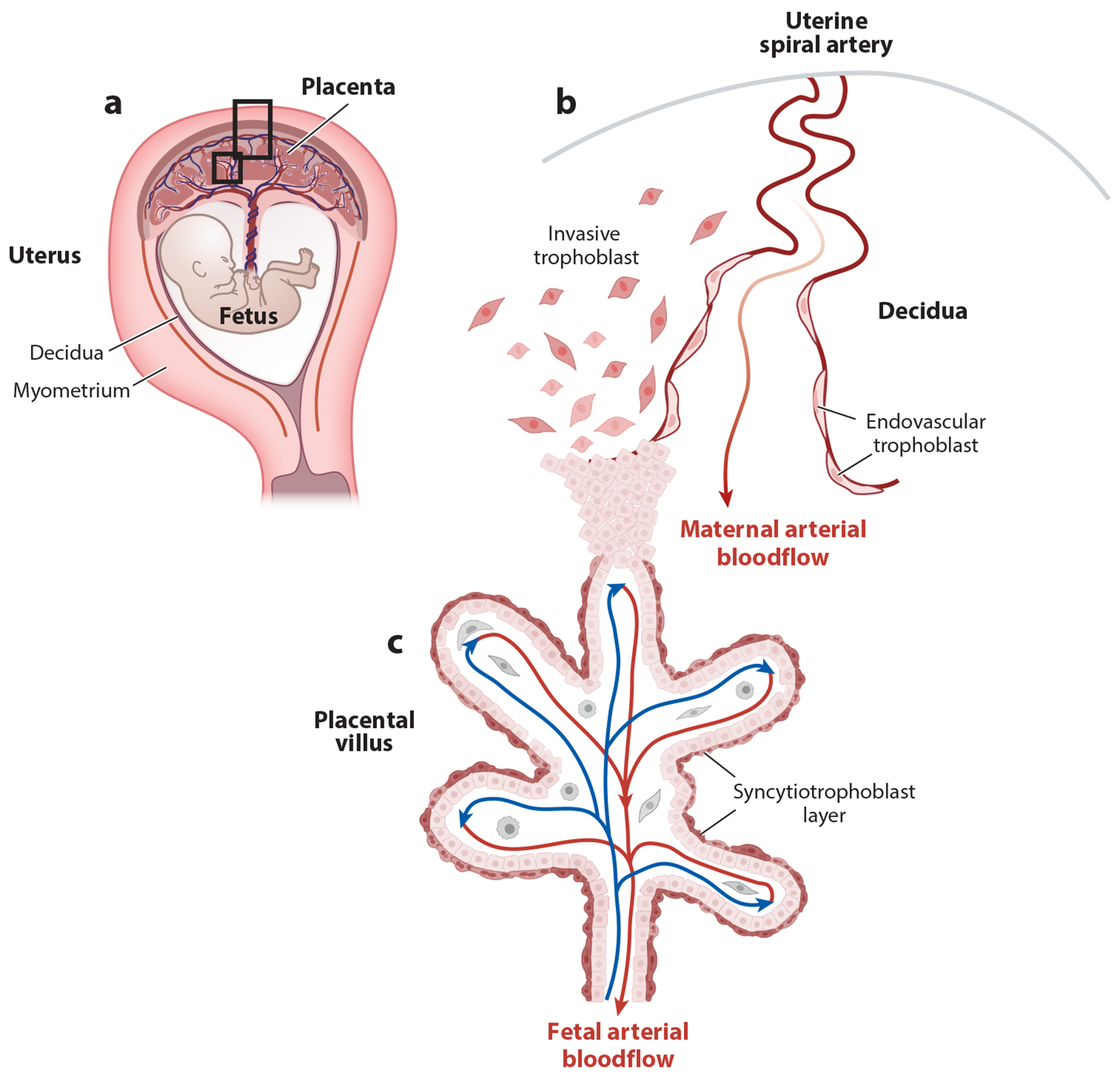
Placental anatomy. (*a*) Diagram of late human gestation shows the placenta and fetus situated within the uterus. The epithelial lining of the nonpregnant uterus (endometrium) transforms into decidua around the time of implantation. Myometrium is the outer, smooth muscle cell layer of the uterus. The placenta invades and is anchored into the decidua. Rectangles highlight areas depicted in panels *b* and *c*. (*b,c*) Microanatomy of (*b*) the interface between uterus and placenta and (*c*) the functional unit of the placenta (villus). Types of trophoblast populations most pertinent to the subject of fetomaternal tolerance are depicted. Some villi anchor the placenta to the decidua, as shown. Invasive trophoblasts migrate to the inner third of the myometrium. Some invasive trophoblasts participate in remodeling of maternal vasculature converting muscularized arterioles into wide-bore, low-resistance vessels lined by endovascular trophoblasts. This process ensures adequate blood flow to placental villi. Placental villi, directly bathed in maternal blood, are the main functional units of the placenta. Villi consist of fetal blood vessels embedded in stroma, enveloped by a layer of fused trophoblast cells (syncytiotrophoblast layer). As discussed in the text, trophoblast antigens enter maternal circulation and local lymphatics, and disruption of the syncytiotrophoblast layer and fetal endothelium can leak fetal blood cell antigens into maternal circulation. Note: mouse placentas (not depicted) bear structural similarities, although they feature shallower invasion and prominent yolk sacs (as discussed in the text). Figure adapted from images created with BioRender.com. Panel *c* adapted from Reference 2.

Fetomaternal tolerance is not accomplished by generalized maternal immunosuppression as was once hypothesized and then adopted as dogma. Instead, fetomaternal tolerance is largely conceptus specific, while maintaining robust B cell and antibody responses to foreign antigen. Antigen-specific fetomaternal tolerance pathways are inextricably linked to the anatomy and function of the placenta, the fetal-derived organ that interfaces directly with maternal immune cells. Briefly, the placenta develops from the outer cells of the blastocyst and invades into the maternal uterus to facilitate gas and nutrient exchange between maternal and fetal circulations. Evolution did not place maternal and fetal blood in direct contact. Rather, small molecules, including maternal immunoglobulin G (IgG) antibodies, transit across placental epithelial cells, which in many types of placentas (including mouse and human) are positioned directly in the maternal bloodstream (Figure 1).

The main placental epithelial cell type is the trophoblast cell. The microanatomy and cellular composition of the uterus-placental interface, and the “shedding” of trophoblast and other fetal cell-derived material into maternal circulation, imply that maternal B cells may encounter conceptus antigens locally in the uterus and systemically in bone marrow (BM) and secondary lymphoid organs. Trophoblasts express many antigens foreign to maternal immune cells (2), including some that provoke antibody-mediated rejection in organ transplant settings (3, 4). Trophoblast antigens that might be recognized by maternal B cells include cell-type-specific antigens, oncofetal antigens, minor histocompatibility antigens, and major histocompatibility (MHC) antigens HLA-C (human leukocyte antigen C) and mouse H-2K/D. Moreover, spillover of fetal blood cells into maternal circulation due to placental microhemorrhages and vascular shearing at delivery exposes maternal B cells to all paternal-derived MHC antigens.

Nearly a century ago, clinicians defined hemolytic disease of the fetus and newborn (HDFN) [also known as Rhesus D (RhD) disease for the antigenic trigger] as a pregnancy complication caused by maternal IgG with specificity for fetal red blood cells (RBCs) (5). At initial observation, one might draw the shocking conclusion that maternal–fetal incompatibility in the RBC surface protein RhD is the sole instance of maternal cellular or humoral-mediated rejection of the conceptus. This raises two very important questions—namely, (*a*) what mechanisms enforce cellular and humoral tolerance to all other paternal-derived alloantigens (a corollary of which is why do they fail in the case of RhD?) and (*b*) are maternal B cells and antibodies involved in other adverse pregnancy outcomes, and, if so, how?

To tackle these questions and discover mechanisms of B cell fetomaternal tolerance, contemporary researchers have taken advantage of clinical observations, human placental and peripheral blood samples, and mouse models that allow for use of modern tools to probe immune responses in ways that are not possible in humans and were not available in decades prior. Below, I summarize data that show that tolerance mechanisms restrain B cells and antibodies from harming the conceptus in healthy pregnancy. Additional research and clinical observations showing that B cells and antibodies play crucial roles in the pathogenesis of adverse pregnancy outcomes beyond HDFN are then described. Included among this considerable body of research is a beautiful series of bench-to-bedside studies that advanced the field’s understanding of how antiphospholipid antibodies mediate adverse outcomes, ultimately leading to a current clinical trial testing whether a targeted biologic agent prevents preeclampsia (6). Throughout, instances where historic literature foreshadowed more recent identification of molecular mechanisms are mentioned, and attractive historic hypotheses that have yet to be comprehensively tested are emphasized.

## CHANGES TO B CELLS IN PREGNANCY

**Figure 2:**
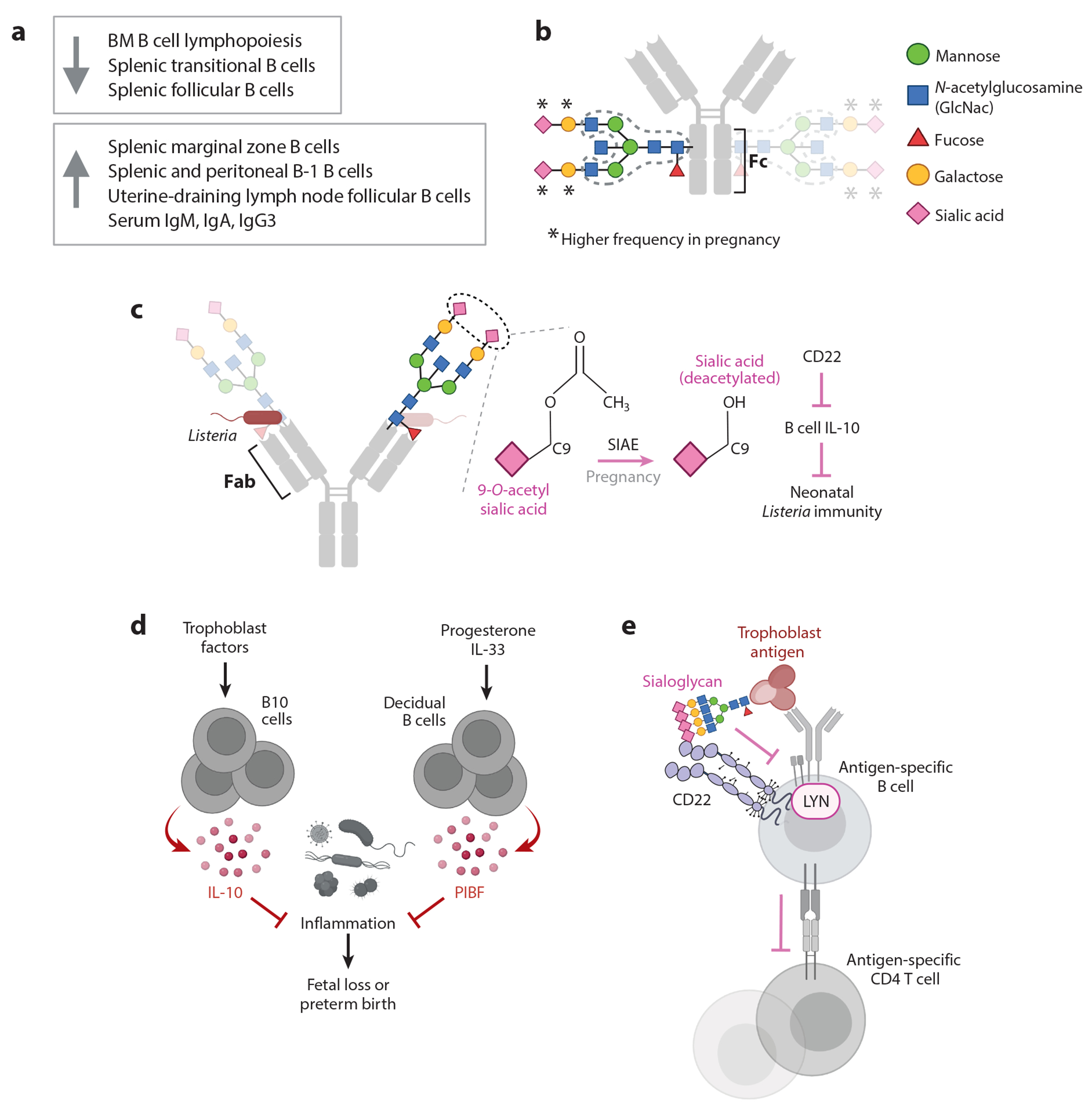
B cell and antibody adaptations to pregnancy. (*a*) Summary of changes in the frequency of B cell subsets and immunoglobulin isotypes in pregnant mice and humans. (*b*) Schematic of the complex, biantennary N-dglycan linked to asparagine 297 residues of an immunoglobulin G (IgG) Fc CH2 domain. Variable amounts of certain sugar monomers (fucose, galactose, sialic acid, and the presence or absence of a “bisecting” GlcNac between the two antennae) are attached to a core structure (*dashed outline*). Roughly 30 different Fc glycoforms have been described. Pregnancy is associated with significant increases in galactose- and sialic acid–containing Fc glycoforms. (*c*) Pregnancy is also associated with changes to the terminal sialic acid residues of antigen-binding fragment (Fab) N-glycans, with functional consequences for pathogen defense in the neonate. Maternal listeria-specific IgG Fab glycans become deacetylated by the enzyme sialic acid acetyl esterase (SIAE). After passive transfer across the placenta, the sialic acids on IgG Fabs interact with CD22 to suppress neonatal B10 cells, which otherwise inhibit the neonatal response to this pathogen. C9 is the ninth carbon of sialic acid, subject to modification by an acetyl group. (*d*) Pregnancy factors, including trophoblast-derived hormones, and interleukin 33 (IL-33) increase the frequency and function of anti-inflammatory B cell subsets. IL-10-producing splenic and uterine B cells, as well as progesterone-induced blocking factor (PIBF)-producing decidual B cells, have been shown to dampen strong maternal inflammation that otherwise triggers early labor. The antigen specificity of these cells is not known. (*e*) Activation of maternal splenic B cells with specificity to sialylated trophoblast antigen is suppressed by coengagement of sialic-acid binding receptor CD22. CD22 requires the Src-family tyrosine kinase LYN for signaling that antagonizes B cell receptor–mediated activation. B cell suppression, in turn, underscores CD4 T cell tolerance to some trophoblast antigens. Involvement of other inhibitory receptor targets of LYN is likely, since responses are significantly more pronounced in *Lyn*^−/−^ versus *Cd22*^−/−^ hosts. Other abbreviation: BM, bone marrow. Figure adapted from images created with BioRender.com. Panel *e* modified from Reference 2.

When compared with the nonpregnant state, various changes in the frequency of B cell subsets and immunoglobulin isotypes occur in pregnant humans and mice (Figure 2a). For most changes, a mechanistic etiology and the physiologic relevance to pregnancy remain unknown. A logical hypothesis is that these changes foster fetomaternal tolerance without significantly compromising defense from pathogens. For example, a large reduction in the generation of new B cells is observed in the BM during mouse pregnancy, commencing at embryonic day 7.5 (E7.5) and maintained postpartum by lactation (7). The decrease in B cell lymphopoiesis, which can be mostly modeled in virgin mice by administration of 17β-estradiol, is characterized by a severe reduction in all interleukin 7 (IL-7)-dependent developmental precursors and reduced proliferation of newly formed, immature B cells (7). This is also reflected in reduced recent BM emigrants peripherally (i.e., transitional B cells) and, to a lesser extent, reduced naive follicular B cells (by ~25%) beginning at approximately E14.5 in the spleen (8). Features of pregnancy besides estrogen that contribute to this phenomenon were hinted at by studies showing elimination of newly formed BM B cells with B cell receptors (BCRs) recognizing placental antigen with high affinity (9, 10). Thus, changes in B cell development may foster fetomaternal tolerance by preserving a temporarily stable BCR repertoire among the naive cell pool.

Pregnancy-associated alterations in mature peripheral B cells include expansion of mouse splenic marginal zone (MZ) B cells, mouse splenic and peritoneal cavity resident B-1 B cells, and increased serum levels of IgM, IgA, and IgG3 in both mice and humans (8, 11, 12). Additionally, the population of mouse splenic CD19^+^CD5^+^CD1d^hi^ B cells, which increases by 50% during normal matings but not in matings between strains with a high frequency of fetal loss, includes B cells that produce IL-10 (13). The role of these cells is elaborated upon in the section below titled B Cells with Anti-inflammatory Properties Safeguard Pregnancy. As primary sources of germline-encoded, polyreactive “natural” antibodies, B-1 and MZ B cells play important roles as first-line defenders before development of an adaptive B cell response. Elevated numbers of these cells during pregnancy may thus serve to bolster immune defense against infections.

Pregnancy is also accompanied by local changes to B cells that reside within and closer to the uterus. B cells comprise ~2% of total uterine leukocytes, but their precise distribution within different tissue layers of the pregnant uterus (decidua and myometrium) and parenchymal versus vascular residency across gestation has not been determined. Still, thorough profiling of total uterine and uterine-draining lymph node (UDLN) B cells across gestation shows a twofold increase in mouse uterine B cells during the peri-implantation period (14). A similar twofold increase in B cells is observed in mid- to late-gestation UDLNs (14). Whether B cell expansion is in response to conceptus-derived antigen versus a non-antigen-specific stimulus has not been assessed. It also remains unclear whether B cells play a role in the expansion of local FOXP3^+^ regulatory T (Treg) cells during the peri-implantation period (15), through MHC-II-mediated antigen presentation, production of IL-10, or transforming growth factor beta (TGF-β).

## CHANGES TO ANTIBODIES IN PREGNANCY

Pregnancy causes shifts in the abundance of complex, biantennary, N-linked glycans attached to the Fc region of IgG (Figure 2b). Changes described thus far include a small but significant increase in glycans containing galactose and sialic acid during the second and third trimesters of human pregnancy (16, 17) and the latter half of mouse pregnancy (18). These changes return to prepregnancy proportions postpartum. Fc glycans dictate IgG-mediated effector functions by impacting affinity for Fc receptors, lectin receptors, and complement component C1q (19). Our understanding of how Fc glycans might influence tolerance to the conceptus or immunity to pathogens during pregnancy is still in its early stages. However, valuable insight has already been gained from examining Fc glycans in pregnant women who suffer from rheumatoid arthritis (RA). RA symptoms improve in the latter half of gestation, only to flare postpartum and then return to prepregnancy levels (20). Notably, pregnancy-associated waning and waxing of RA symptoms in both humans and mouse models closely correlates with changes in frequency of total serum IgG1 with highly galactosylated Fc glycans (16, 18). Mechanistically, a high frequency of galactose-containing glycans is essential for mediating anti-inflammatory effects of IgG1 immune complexes (21). Specifically, galactosylated Fc regions of IgG1 can inhibit complement-mediated activation of myeloid cells by engaging inhibitory signaling via their high affinity for Fc gamma receptor IIb (FcγRIIb) and Dectin-1 (22). Beyond their potential role in autoimmunity, understanding if and how Fc glycans contribute to healthy pregnancy is an important and open area of research. It is certainly possible that altered IgG glycans contribute to adverse pregnancy outcomes marked by heightened complement activation and placental inflammation, as discussed in the second half of this review.

N-linked glycans decorate the antigen-binding fragment (Fab) of approximately 15% of serum IgG, and these glycans are also subject to pregnancy-associated modification (23). Changes include a very small increase in frequencies of sialic acid–containing Fab glycans (17). While very few N-glycosylation consensus motifs are encoded in germline Fab V-region coding sequences, they arise during somatic hypermutation (24, 25). B cells with glycosylated Fab are thus subject to selective pressure in germinal centers (GCs). It has been suggested that Fab glycans might reduce affinity for cognate self-antigen and thus enable maintenance of autoreactive B cells (24). Perhaps maternal Fabs undergo glycan modifications to reduce undesired reactivity toward conceptus antigens, but this is purely speculative. Of note, older literature concluded that pregnant serum might contain paternal antigen-specific IgG with glycans attached to only one of two Fab arms (26, 27) (so-called asymmetric or hemiglycosylated IgG). Hemiglycosylation would impair the function of such antipaternal IgG by preventing aggregation into immune complexes (28); however, it is unclear how asymmetry arises.

An important job of the placenta, described further below, is the passive transfer of maternal IgG to the fetus (29). Recent work shows that a pregnancy-associated glycan modification to the Fab region of IgG in maternal serum alters function to significantly enhance antipathogen immunity in neonates (30) (Figure 2c). In a mouse model of neonatal *Listeria monocytogenes* (*Lm*) infection, researchers observed that Fab glycans on listeria-specific IgG generated in response to vaccination of the mother before pregnancy contained *O*-acetylated terminal sialic acid residues (30). The acetyl modification of sialic acid prevents recognition by sialic acid binding receptors, such as CD22, and is thus functionally significant. During pregnancy, the preexisting *Lm*-specific IgG became enzymatically deacetylated by sialic acid acetyl esterase, which was upregulated at the transcript level in maternal splenocytes. In the neonate, deacetylated anti-*Lm* IgG conferred protection in a mechanism involving CD22-mediated inhibition of B10-like B cells, which otherwise suppressed immunity to *Lm* (30). This work raises many interesting questions, including which maternal IgG antibodies undergo deacetylation and how this choice is made. Perhaps additional maternal or conceptus-derived sialylated proteins are similarly deacetylated, thus increasing recognition by CD22 to suppress self- or conceptus-specific B cell reactivity.

At present, I can only speculate about molecular mechanisms that drive glycan alterations in pregnancy. Of course, the complex physiology of pregnancy is a likely general factor driving changes to IgG glycans. Pregnancy-associated changes to metabolism could underscore availability of sugar monomers and intracellular glycan synthesis pathways. Additionally, pregnancy hormones and cytokines may be partially responsible for initiating changes to glycosylation. Older in vitro work shows that B cell hybridomas cultured in the presence of progesterone and IL-6 increased expression of oligosaccharyltransferase (31), the enzyme complex that initiates N-glycosylation in the endoplasmic reticulum. Progesterone-induced blocking factor (PIBF) also altered the glycan pattern on hybridoma-derived IgG (32). However, it is unlikely that the increase in sialic acid and galactose-containing Fc glycoforms is solely accounted for by pregnancy hormones, since these changes are not observed in pseudopregnant mice (18). A key unknown is to what degree modifications are made on circulating serum IgG versus at the level of antibody-producing plasma cells. Intriguingly, recent work outside the context of pregnancy supports a model whereby terminal sialic acids are added to Fc glycans not by plasma cells, but by endothelial cells after release from plasma cells (33–35). Support for this model comes from the observation that IgG sialylation was intact in mice lacking β-galactoside α2,6-sialylatransferase 1 (*St6gal1*) in B cells (34) but significantly decreased in mice globally deficient in neonatal Fc receptor (FcRn) expression (33). FcRn^+^ endothelial cells, well documented for their role in the recycling pathway that prolongs IgG serum half-life, attached terminal sialic acid to IgG glycans in vitro (33). With regard to pregnancy, might FcRn^+^ placental cells not only transport maternal IgG but also modify glycans? Finally, apart from IgG glycans, future investigation may reveal pregnancy-associated alterations in glycosylation of other proteins with crucial immunologic roles.

## B CELLS WITH ANTI-INFLAMMATORY PROPERTIES SAFEGUARD PREGNANCY

Research conducted over the last decade unveiled roles for anti-inflammatory B cells in suppressing infection-induced maternal inflammation that might otherwise trigger fetal loss (Figure 2d). As mentioned above, a 50% increase in CD19^+^CD5^+^CD1d^hi^ B cells is observed at E14.5 in healthy mouse gestation (13). A similar increase in the frequency of human peripheral blood CD19^+^CD24^hi^CD27^+^ B cells is observed in the first trimester (36). Both populations contain cells that produce IL-10 in vivo. These anti-inflammatory cells have been variously referred to as B10 B cells or regulatory B cells. Here, for consistency, I use B10 but note that elaboration of factors besides IL-10 may dominate their function in some contexts. Stimuli for production of IL-10 include soluble and perhaps contact-dependent trophoblast-derived factors (37, 38). Human primary B cells, when cultured with human chorionic gonadotropin, and primary mouse B cells stimulated with CD40L plus CpG and cocultured with trophoblast cell lines or placental explants both exhibited heightened production of IL-10 (37). In terms of functionality, B10 cells mitigate fetal loss caused by inflammation. In models of infection-induced preterm birth, pregnant mice lacking B cells or IL-10 experienced fetal loss at lower lipopolysaccharide (LPS) doses compared with wild-type pregnant mice (39, 40). Although the specific cellular targets of IL-10 in this model remain unidentified, Treg cells are a likely target given the lack of splenic Treg cell expansion in B cell–deficient pregnant mice (40). The expansion of Treg cells is, to some extent, in response to antigens derived from the conceptus (41), but it remains unknown if the B10 population includes clones that recognize conceptus antigens.

Despite their low numbers, uterine B cells contribute to healthy pregnancy by restraining excess inflammation that might otherwise trigger labor pathways in late gestation or hinder placental development in early gestation. The development of anti-inflammatory B cells in the human decidua relies upon local progesterone and IL-33, which together induce B cells to produce immunosuppressive PIBF (42). PIBF is multifaceted but functions primarily to dampen acute inflammation and innate cell cytotoxicity. Compared with full-term decidua samples, lower levels of PIBF were observed in samples from spontaneous preterm birth (42), supporting the hypothesis that dysregulation in the generation or maintenance of PIBF-producing decidual B cells contributes to inflammation-induced labor. Mouse uterine B cells appear to adopt an antiinflammatory phenotype even earlier in gestation. Compared with B cells from the nonpregnant uterus, uterine B cells from E5.5 produced elevated levels of IL-10 in response to LPS stimulation in vitro (14). Pregnant mice lacking B cells or B cell–derived IL-10 showed diminished conceptus sizes, reduced placental diameters, and small increases in uterine arterial resistance, compared with pregnant wild-type mice (43). Thus, although B cells are not required for pregnancy in mice or humans, certain B cell subsets appear to promote homeostasis and placental development by regulating inflammation.

## ANTIBODIES AND THE PLACENTA

Despite quantitative and qualitative changes in B cells and antibodies during pregnancy, overall effectiveness of maternal humoral immunity against foreign antigens remains generally intact. Assessments of humoral quality following infection and immunization reveal few meaningful differences in antibody titer and neutralization capacity across pregnant populations and pathogens (44–48). These protective antibodies, transferred from maternal to fetal circulation by the placenta in utero, as well as from breast milk postpartum, are vital to neonatal immune defense and early life development.

Passive transfer in humans occurs via binding of IgG to FcRn on surface microvilli of the syncytiotrophoblast layer, transcytosis, and then additional undefined mechanisms of transit across fetal endothelial cells. This has been comprehensively reviewed elsewhere (49, 50). Here, I briefly highlight mechanisms of passive transfer in mice. While vestigial in humans, yolk sac endoderm surrounds rodent embryos throughout gestation and provides nutritional support (51). Molecules from maternal circulation and endometrial glands transit across yolk sac endodermal cells, through stroma containing macrophage-like cells, and into fetal vitelline vasculature. Curiously, yolk sac endoderm is the site of FcRn expression in the mouse (52). When mice with heterozygosity for the FcRn gene reproduce, FcRn null neonates lack serum IgG and heterozygous pups have 50% lower levels of serum IgG, compared with wild-type littermates (52). Thus, while the location of FcRn differs between species, FcRn appears to serve the same function. Interestingly, the efficiency of IgG placental transfer is affected by antibody subclass (53) and Fc glycosylation (54) in a manner that cannot be explained by differences in FcRn affinity. Preferential transfer then might depend upon differing affinity for additional Fc receptors (FcRs) present on stromal cells, macrophage or macrophage-like cells, or fetal endothelial cells (55, 56).

Oft-overlooked historic literature suggests that the placenta may not only supply protective antibodies to the fetus but also limit transfer of paternal-specific antibodies. Experiments conducted in the 1970s demonstrated IgG strongly bound to the basement membranes of villus trophoblasts in term human placentas (57, 58). Such IgG, eluted and purified, was shown to react with villus trophoblasts by immunohistochemical staining and to inhibit mixed lymphocyte reactions (57). Mouse studies revealed that IgG appeared to be bound to the surface or within the cytoplasm of early gestation trophectoderm and trophoblasts at the invasive edge of the ectoplacental cone (59) and, later, to trophoblast giant cells, yolk sac endoderm, and the fetus (60). As mouse trophoblasts are not involved in passive transfer, the role of bound IgG, theorized to involve inhibition of cellular reactivity to placental antigens (57), remains unknown. The hypothesis that antipaternal antibodies are trapped and destroyed by placental cells that express cognate paternal antigen (61) was also evaluated in historic experiments. Injected into pregnant mice, radiolabeled allospecific anti-H-2 antibodies [or F(ab′)_2_ fragments] resulted in accumulation and apparent degradation within cognate antigen-expressing placentas, without a corresponding accumulation in fetuses (62–64). Similar findings were reported for naturally occurring antipaternal antibodies recognizing paternal strain antigens in allogeneic matings (65). However, experiments conducted a decade later, using higher dosing of a distinct antibody clone, were unable to replicate this phenomenon (66). Consequently, examination of whether placentas transfer versus degrade antipaternal IgG remains a subject awaiting contemporary exploration.

## PLACENTAL ANTIGEN-SPECIFIC B CELL RESPONSES

The placenta is an abundant source of antigens that might be recognized by maternal B cells (2). Paternal HLA-C in humans and H-2K/D in mice and a plethora of minor histocompatibility antigens may all in theory undergo cell-free or vesicular transport from the placenta to UDLNs and spleen. Evidence thus far supports the hypothesis that tolerance checkpoints prevent the generation of robust, high-affinity, antiplacental B cell responses. Studies from the 1990s identified a developmental checkpoint that prevents introduction of new placental antigen-specific B cells into the peripheral repertoire (9, 10). These first studies used female mice carrying the 3.83 BCR transgene, which recognizes H-2K^k^ with high affinity. When mated to males homozygous for cognate H-2K^k^ antigen, but not to syngeneic or third-party haplotype male mice, a 50% reduction in immature B cells was observed in maternal BM, spleen, and blood (10). This reduction began at mid-gestation concomitant with when the placenta is bathed in maternal blood, persisted for several days postpartum, and was not observed in UDLNs (10), suggesting removal of newly formed clones at sites where final maturation of B cells takes place. Indeed, this is a documented tolerance checkpoint for elimination of self-reactive clones, so it is not surprising that high-affinity, placenta-specific clones are subject to a similar fate. However, the supraphysiologic precursor frequency of B cell clones in these transgenic mice, along with the high receptor affinity, likely inflated the degree to which deletion occurred. In fact, when 3.83 BCR transgenic females were mated to males homozygous for the lower-affinity H-2K^b^ antigen with expression restricted to trophoblast giant cells, deletion was apparent only in BM (9).

More recently, it was discovered that placental antigen-specific mature splenic B cells are rendered unresponsive in a mechanism involving antigen glycosylation and requiring at least some of the B cell machinery involved in tolerance to self-antigen (67) (Figure 2e). These experiments were performed using a model whereby half of the litter inherits a paternal transgene directing cell-surface expression of full-length chicken ovalbumin (OVA) (68), a model antigen commonly used to delineate mechanisms of tolerance and immunity in vivo. While the transgene drives ubiquitous expression, OVA is most highly expressed on trophoblasts in direct contact with maternal blood (68). Like other trophoblast proteins, OVA was released via an unknown mechanism in cell-free form into maternal circulation beginning at mid-gestation. OVA-specific follicular B cells in maternal spleen became aware of shed trophoblast OVA several days thereafter. Remarkably, even in the presence of adjuvants and OVA-specific CD4T cells, OVA-specific B cells resisted expansion and activation (67). Comparison of shed trophoblast OVA and chicken OVA showed that while both proteins are decorated with N-glycans, N-glycans with sialic acids were identified only on trophoblast OVA. Moreover, trophoblast OVA was more heavily sialylated than OVA from nonplacental organs (e.g., adult skin, liver, even fetus proper) in OVA transgenic mice. This was notable since sialic acids can serve as “self-associated molecular patterns” that suppress immune responses by engaging cell-surface sialic acid–binding immunoglobulin-like lectins (Siglecs) (69). CD22 is one such receptor that shows largely B cell–specific expression and requires LYN tyrosine kinase for signaling, which antagonizes B cell activation (70). Strikingly, B cells exposed to trophoblast OVA were no longer rendered tolerant in *Cd22*^−/−^ mice, and the B cell response was even more unleashed in *Lyn*^−/−^ mice (67).

Hence, results from this work indicated that Siglec-mediated recognition of sialoglycans on trophoblast proteins suppresses activation and expansion of naive placental antigen-specific B cells. It is also possible that antigen sialoglycans are involved in the developmental deletion of immature B cells since administration of experimentally sialylated antigens results in deletion of antigen-specific B cells in vivo (71). Remarkably, the significance of sialoglycans in fetomaternal tolerance was foreshadowed in experiments conducted more than 50 years ago. This literature contains evidence that immunogenicity of placental homogenates increased after removal of sialic acids by treatment with sialidases (72). Accordingly, it is possible that sialoglycans also underscore fetomaternal T cell tolerance via Siglec-mediated inhibition of B cells and other antigen-presenting cells that process and present placental antigens. While the field has yet to directly interrogate B cells with specificity to antigens beyond the few highlighted here, a similar sialoglycan signature is observed on placental and fetal-derived proteins identified in plasma from pregnant humans and mice (67). Thus, this mechanism could extend quite broadly. It is also unknown if changes in placental glycosylation contribute to pregnancy complications, although intriguingly, sialic acid–related genes were reported to be differentially regulated in placentas from women with preeclampsia (73, 74).

## ANTIPATERNAL ANTIBODIES IN MICE AND HUMANS

Antibodies to paternal antigens emerge in successful mouse pregnancy. As early as the 1960s, reports documented the presence of alloantibodies in multiparous mice after allogeneic pregnancies (75). Antipaternal antibodies were then carefully investigated in a clever series of experiments two decades later (65, 76–79). These studies found that antipaternal antibodies variably emerged by the end of a first pregnancy (i.e., antibodies were detectable only in some mice and only in some strain mating combinations) and titers were boosted in subsequent pregnancies (77). Such antibodies were predominantly IgG1 subclass and did not fix complement (76); however, the identity of their antigenic targets or even the cellular source of antigen remains unknown. Interestingly, vaccination of virgin mice with crude placental extracts generated non-complement-fixing IgG1 antibodies, while fetal extracts generated complement-fixing IgG2 antibodies (78). This raises the possibility that tissue-specific glycosylation patterns trigger different types of antibody responses to placental versus fetal antigen.

The inability of these antipaternal antibodies to fix complement, which can be considered a tolerance mechanism, is surprisingly not a requirement for healthy pregnancy. More recent work in an allogeneic OVA pregnancy model detected a predominance of antipaternal IgG2c (80), which can in theory bind C1q to initiate the classical complement pathway. Moreover, pregnancy is successful in female mice immunized to paternal antigens prior to mating, a phenomenon originally noted in 1953 and reproduced several times since (9, 79, 81). We still lack mechanistic insight into how the placenta handles these antibodies. Placental trophoblasts are well protected from spontaneous complement activation via expression of complement regulatory proteins, including membrane cofactor protein (MCP) and decay accelerating factor (DAF) in humans (82) and CR1-related gene/protein Y (Crry) in mice (83)—perhaps these also protect from antibody-mediated classical complement pathway activation. Trophoblast complement regulatory mechanisms must be quite strong, since high levels of complement-fixing antipaternal IgG generated by immunization before pregnancy were then boosted by exposure to placental antigen at mid-gestation, again with no apparent adverse effect on pregnancy outcomes (79).

Antibodies to paternal antigens also emerge in successful human pregnancy. These were originally discovered by clinicians who noticed that multiparous women frequently exhibited febrile reactions following postpartum blood transfusions (84). The reactions were linked to anti-leukocyte antibodies present in maternal serum. Paternal HLA-specific IgG antibodies develop in more than 50% of first pregnancies and are even more prevalent in multiparous women (85, 86). Algorithmic assessment of triplet amino acid residues in HLA proteins shows that alloantibodies are more likely to arise when there is a higher degree of paternal and maternal sequence mismatch, as might be expected (87). There is an additional correlation between the number of paternal HLA-derived peptides algorithmically predicted to be presented on maternal MHC-II and the probability of developing anti-HLA antibodies (88). This suggests a role for CD4 T cell help in the generation of antipaternal antibodies. Additional biologic and genetic factors may modify the risk of generating anti-HLA antibodies in pregnancy and affect their titer and complement-fixing capacity, with research thus far suggesting roles for soluble HLA-G levels and IL-6 levels (86). However, mechanistic research on how these factors could uniquely affect antiplacental antibody development, without suppressing responses to foreign antigen, is lacking.

How plasma cells specific to paternal antigens emerge is not known. Work in the OVA pregnancy mouse model suggests that rather than a GC response, memory B cells and antipaternal antibodies arise from an alternate trajectory but still require interaction with cognate CD4 T cells (80). Future studies will have to decipher how CD4 T cells, which seemingly develop tolerance to paternal antigens (41, 67), participate in this response. Additional important features such as antibody affinity and specificity of non-MHC target antigens also require definition. Classically, a GC response involving cognate antigen-specific follicular helper T (Tfh) cells produces long-lived memory B cells and plasma cells harboring somatically mutated, high-affinity antibodies. There are many instances, notably in lupus models and in response to certain pathogens, where responses occur outside of GC follicles (89). Such extrafollicular responses can sustain some level of affinity maturation and give rise to long-lived memory cells and plasma cells, which could be sources of antipaternal antibodies.

The existence of antibodies and memory B cells with specificity for paternal antigens threatens transfusion and transplant success in patients with a history of prior pregnancy. As reviewed previously (90), women with anti-HLA antibodies experience longer wait times for organs, and prior pregnancy influences gender-based differences in outcomes (91). Recent work in mice identifies paternal antigen-specific B cells and antibodies as central drivers of rejection of an antigen-matched organ transplant (80). Using a clinically relevant regimen that tolerized virgin wild-type mice into accepting F1 strain heart grafts, wild-type mice showed accelerated rejection of F1 grafts after allogeneic pregnancy. Remarkably, μMT strain mice, which lack B cells and antibodies, accepted F1 heart grafts after allogeneic pregnancy, even without the tolerizing regimen (80). This aligns with earlier findings on T cell fetomaternal tolerance demonstrating pregnancy induction of tolerogenic CD4 T cell memory and reduction in reactivity of paternal antigen-specific CD8 T cells. It is unclear exactly how pregnancy-induced antibodies and memory B cells provoked graft rejection. However, it was noted that transfer of serum alone from postpartum wild-type mice or B cells alone from postpartum mice that lack serum antibodies were each capable of precipitating graft rejection in postpartum μMT mice (80). While we await additional mechanistic answers, these findings already raise the exciting possibility that selective elimination of B cells and alloantibodies might foster acceptance of partner-matched organs in multiparous women.

Incredibly, we lack compelling evidence to support the notion that antipaternal antibodies, which arise during healthy pregnancy and compromise subsequent transplant, either harm or contribute positively to fetomaternal tolerance. Perhaps they are best conceptualized as an “epiphenomenon of any pregnancy,” as was previously suggested (92, p. 94). However, as detailed in the section below titled Humoral Mechanisms of Rejection, exceptional fetal and placental antigens elicit pathogenic maternal antibody responses or are targeted by preexisting maternal autoantibodies. Both situations unequivocally mediate harm to pregnancy. Moreover, as I also describe below, it is possible that antipaternal antibodies participate in several enigmatic placental pathologies associated with significant adverse outcomes. A better understanding of how pregnancy can proceed unharmed in experimental situations where robust cytotoxic antipaternal antibodies are present could shed light on where along the tolerance pathway breakdowns occur and how this causes disease.

## HUMORAL MECHANISMS OF REJECTION

**Figure 3:**
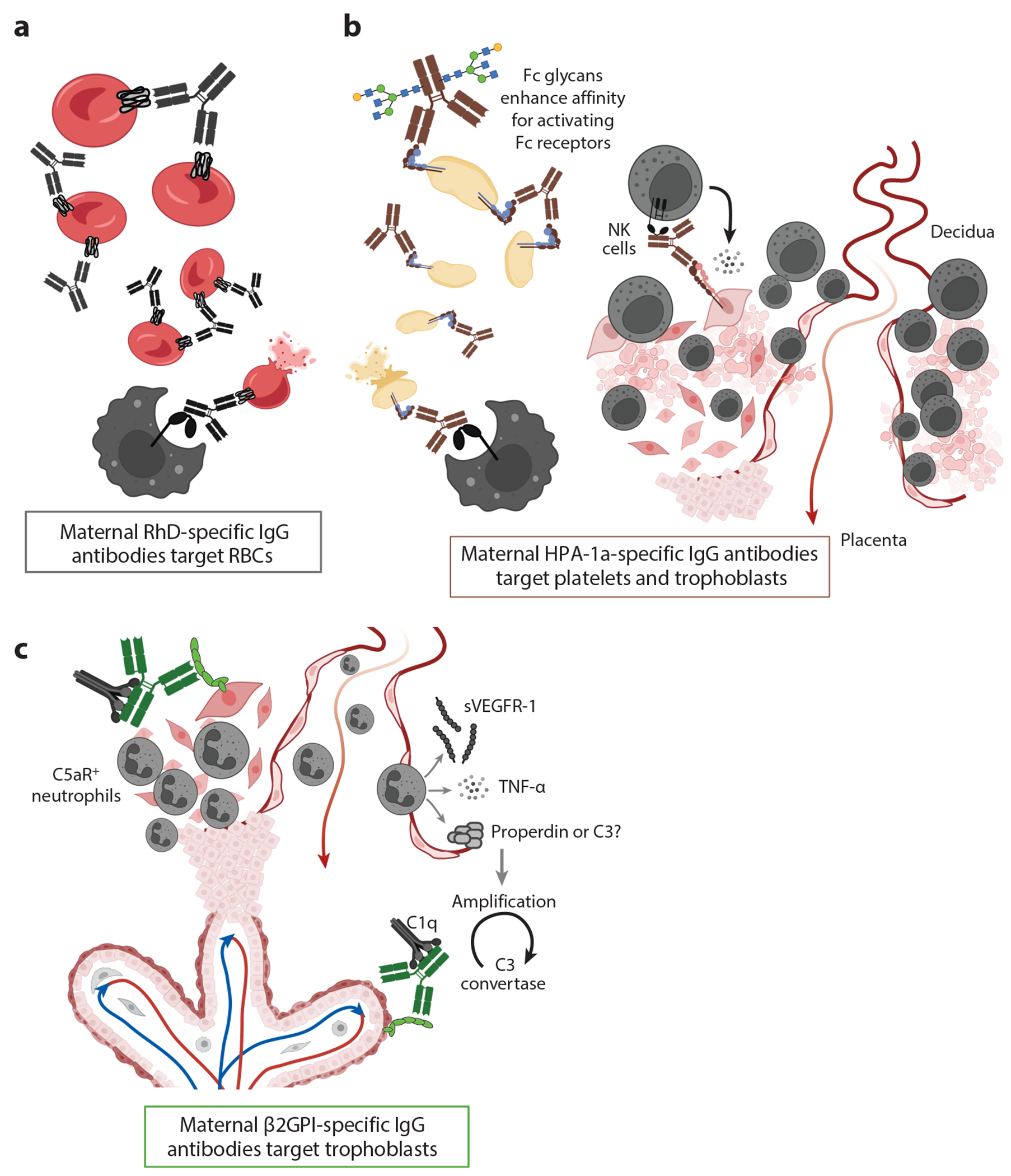
Pathogenesis of maternal antibody-mediated damage to the placenta and fetus. (*a–c*) The three scenarios where breakdown of B cell fetomaternal tolerance results in adverse pregnancy outcomes. (*a*) Hemolytic disease of the fetus and newborn (HDFN) is mediated by maternal immunoglobulin G (IgG) specific for paternal-derived fetal red blood cell (RBC) antigens. After passive transfer across the placenta, anti-RBC IgG antibodies mediate extravascular hemolysis of fetal RBCs, as shown. Historically the most common etiology for HDFN was maternal–fetal incompatibility in Rhesus D (RhD) antigen. Note that the specific humoral pathways, including the role of CD4 T cell help, leading to the generation of allospecific IgG in pregnancy have not yet been experimentally defined. (*b*) Fetal and neonatal alloimmune thrombocytopenia (FNAIT) is commonly mediated by maternal IgG specific for paternal-derived human platelet antigens (HPAs). The HPA-1a antigen resides in β3-integrin, which is expressed on platelets, endothelial cells (not shown), and trophoblasts. Thus, antibody-mediated damage is incurred by multiple fetoplacental cell types. Antibody-coated platelets are destroyed via extravascular hemolysis, and antibody-coated trophoblasts become targets for maternal natural killer (NK) cells. Engagement of NK cell FcγRIIIa (Fc gamma receptor IIIa) may be enhanced by IgG Fc glycans with decreased core fucose. Activation of NK cells and release of perforin may provoke trophoblast apoptosis. (*c*) Antiphospholipid autoantibodies, including β2 glycoprotein I (β2GPI)-specific IgG, elicit adverse pregnancy outcomes largely due to their ability to activate complement and cause inflammation in the placenta and decidua. β2GPI-specific IgG antibodies initiate disease by activating the classical pathway of complement. Anaphylatoxin C5a recruits neutrophils, which elaborate factors to trigger the alternative pathway and amplify the cascade. Neutrophils, and perhaps other innate immune cells, also produce tumor necrosis factor alpha (TNF-α) and antiangiogenic factors such as soluble vascular endothelial growth factor receptor-1 (sVEGFR-1), which can disrupt remodeling of spiral arteries and maternal vascular perfusion of the placenta. Figure adapted from images created with BioRender.com.

The remainder of this review explores uncommon scenarios where research and clinical evidence clearly show that breakdown of B cell fetomaternal tolerance results in adverse pregnancy outcomes (Figure 3). An attempt is made to highlight and speculate upon features that distinguish the B cell and antibody responses detailed below from the harmless responses described above. The Supplemental Material explores how maternal humoral immunity may mechanistically contribute to three additional placental pathologies that are associated with adverse outcomes but otherwise poorly understood.

## HEMOLYTIC DISEASE OF THE FETUS AND NEWBORN

Written descriptions of a condition matching the disease we now call HDFN trace back to 400 BC (as reviewed in 93). It was then that Hippocrates used the term fetus carnosus to describe still-born fetuses with an edematous appearance. Many centuries later, clinicians established HDFN as a disease caused by maternal IgG antibodies that recognize paternal alloantigens on fetal RBCs and elicit RBC destruction (94, 95). Most cases involved women who lacked the gene for RhD antigen and carried pregnancies with paternal inheritance of the RhD allele (96). Exposure of maternal B cells to RhD^+^ fetal RBCs occurs in late gestation, when minor disruptions in the syncytiotrophoblast layer and fetal endothelium leak fetal blood into maternal circulation, and again at delivery when tearing of fetal vessels results in larger quantities of fetomaternal hemorrhage. During subsequent pregnancies, maternal RhD-specific IgG antibodies are passively transferred across the placenta and bind to and mediate destruction of RhD^+^ fetal RBCs. Severity of disease ranges widely. More severe anemia results in high-output fetal cardiac failure leading to an accumulation of interstitial fluid and the edematous appearance that was historically noted (93). Prior to the development of an immunotherapy that prevents generation of maternal RhD-specific IgG, clinically significant sensitization occurred in approximately 16% of pregnancies where RhD^neg^ women carried RhD^+^ fetuses (96). Estimates suggest that HDFN used to affect approximately 1% of all pregnancies and was thus a major driver of fetal and neonatal morbidity and mortality (97).

The specific humoral pathways and cell types leading to the generation of RhD-specific IgG in pregnant women have not been experimentally defined. It is reasonable to hypothesize that fetal RBCs are engulfed by maternal splenic dendritic cells, which process and present peptides on MHC-II, providing initial activation of alloantigen-specific CD4 T cells. Alloantigen-specific B cells also interact with, process, and present peptides from RBC alloantigens on MHC-II and could subsequently receive survival signals from Tfh cells in GCs or CD4 T cells in an extrafollicular location. Either trajectory could generate long-lived antibody-producing plasma cells and memory B cells. Older work shows that Rh blood group–specific IgG antibodies rarely fix complement (98). Thus, it is presumed that after anti-RBC IgG antibodies are passively transferred across the placenta, they bind fetal RBCs, destruction of which proceeds via FcR-mediated removal by myeloid cells of the reticuloendothelial system in the fetal spleen and liver (Figure 3a).

RBC antigen systems do not naturally cause transfusion reactions in mice, and obtaining transgenic surface expression of the genetically complex human Rh antigens in mice has proven to be difficult (99). Ten years ago, however, researchers circumvented these challenges and successfully modeled HDFN by mating wild-type females to male mice carrying a single copy of the human *KEL2* gene under the beta-globin promoter (100). Notably, KEL, a polymorphic human RBC transmembrane glycoprotein implicated in rare HDFN cases, is absent in mice. In the mouse model, KEL was expressed on fetal RBC precursors, KEL^+^ fetal RBCs were detected in maternal circulation after delivery, and maternal anti-KEL IgM and IgG antibodies (of all subclasses) were variably detected in mothers after two KEL pregnancies (100). Pathology became evident during the third pregnancy and was manifested by litters with markedly fewer than 50% KEL^+^ pups at birth and anemia in KEL^+^ pups that did survive.

Much is known about mechanisms of alloimmunization to blood transfusion in nonpregnant mice and humans (101). Aside from the observation that pathology is abrogated by maternal B cell and antibody deficiency, additional features that exacerbate or attenuate generation of KEL-specific IgG antibodies and modulate their effector functions have not been directly studied in the setting of pregnancy. The complex physiology of pregnancy, including the global changes in maternal B cells and IgG glycans, and distinct characteristics of fetal antigen and mode of exposure, implies that mechanisms may be pregnancy specific. For instance, there is a noteworthy elevation in serum C3 levels during pregnancy (102). Considering that C3 exerts complex regulation over alloantibody formation to transfused RBCs in nonpregnant mice (103), it will be important to investigate mechanisms specifically in the pregnant host.

Determining the exact cellular and molecular pathways by which fetal RBC antigens elicit HDFN could thus illuminate important facets of fetomaternal tolerance. It is possible that inherent features of RhD foster its immunogenicity and help to explain why this alloantigen results in pathology while other paternal antigens rarely do. For example, among the cell surface proteome, RhD stands out for its distinctiveness in lacking glycans (104). Consequently, this antigen would not be able to engage the suppressive sialic acid/Siglec axis that facilitates maternal B cell tolerance. More broadly, might some conceptus-derived antigens elicit GC responses while others elicit extrafollicular responses? Is this dependent upon the quality of T cell help (i.e., Tfh versus lack thereof)? Clinical observations raise further questions, particularly with regard to the fact that the incidence of clinically relevant HDFN in cases of genetic mismatch is only 16%, but it is unknown where along the path from sensitization to IgG effector function that tolerance dominates. Are some pregnancies protected because fetomaternal hemorrhage results in antigenic doses too low to elicit responses? Or is there a pathway that generates active B cell tolerance to fetal RBCs in the at-risk women who fail to develop anti-RhD IgG? At present, circumstantial evidence suggests that maternal/fetal ABO incompatibility decreases the risk of the sensitization to RhD, raising the possibility that preexisting ABO antibodies facilitate so-called antibody-mediated immunosuppression (AMIS) (105).

AMIS is a phenomenon whereby experimental introduction of antigen-specific IgG in the absence of cellular immunity prevents de novo formation of humoral responses in the recipient (106). Historic studies in the 1960s showed that administration of antisera to a non-self-antigen prior to vaccination sometimes blocked generation of immune memory in the recipient (107, 108). Based upon this knowledge, the first immunotherapy was created to prevent RhD immunization in pregnancy. Pooled IgG from sera of RhD-antigen-sensitized RhD^neg^ donors was enriched for antibodies with reactivity to RhD and formulated for intramuscular injection and remains the standard of care today. The current protocol involves two doses of 300 μg of polyclonal anti-RhD IgG, first at 28 weeks of gestation and again within 72 h of delivery (97).

Despite now decades of clinical experience with anti-RhD prophylaxis, there is lack of consensus on exactly how this prevents formation of RhD-specific IgG in at-risk pregnancies. Unfortunately, this lack of understanding appears to have hampered the development of a monoclonal anti-RhD IgG to replace pooled donor IgG. The term AMIS itself is somewhat misleading, since in some instances preexisting antibodies can mediate enhancement of active immunity rather than suppression (109). For many years, the prevailing theory was that preexisting RhD-specific IgG prevented maternal B cell responses by mediating extravascular clearance of fetal RBCs prior to any interaction with B cells. However, studies testing prophylaxis with monoclonal antibodies showed poor correlation between RBC clearance and effectiveness (106, 109). Research in nonpregnant mice given RBC transfusions also argues against a role for active tolerance induction in antigen-specific B cells via engagement of the inhibitory receptor Fc*γ*RIIb by immune complexes (110, 111). Instead, the dominant mechanism of AMIS appears to be antigen modulation, which requires recipient C3 and Fc*γ*Rs to presumably result in extraction of alloantigen from the RBC (trogocytosis) (112). Perhaps pooled anti-RhD IgG engages a variety of suppressive pathways, and research to identify these should consider prioritizing the use of pregnant, rather than nonpregnant, animal models for reasons outlined above. Nevertheless, anti-Rh(D) IgG as currently produced and used has been incredibly successful: The incidence of HDFN in at-risk pregnancies treated with anti-Rh(D) IgG has fallen to below 1% (96).

## FETAL AND NEONATAL ALLOIMMUNE THROMBOCYTOPENIA

Fetal and neonatal alloimmune thrombocytopenia (FNAIT) is caused by maternal IgG antibodies, which recognize paternal-derived human platelet antigens (HPAs) on fetal platelets. HPAs comprise approximately 35 antigenic determinants present on exposed regions of platelet transmembrane glycoprotein complexes (113). FNAIT is relatively rare and affects between 1 in 1,000 and 1 in 2,000 pregnancies (114). Morbidity ranges widely from mild to severe fetal thrombocytopenia, the latter of which risks triggering intracranial hemorrhage and stillbirth. Another complication is low birth weight (115), which most likely results from placental dysfunction. Unlike HDFN, where the placenta is unharmed, maternal anti-HPA IgG might damage placental cells that share expression of the antigen, as elaborated below. Additionally, unlike HDFN, there is currently no screening program or prophylactic therapy to prevent the formation of anti-HPA IgG. To prevent recurrence of severe FNAIT in a second pregnancy, current regimens include generalized maternal immunosuppression with intravenous immunoglobulin (IVIg) and prednisone (116).

Research on mechanisms of FNAIT has focused on the HPA-1a antigen located in β3-integrin (117–120). β3-integrin is a component of the fibronectin receptor, highly expressed on platelets, as well as the vitronectin receptor, expressed on some trophoblasts (120) and fetal endothelial cells (119). Maternal β3-integrin-specific IgG antibodies not only trigger fetal thrombocytopenia but also demonstrate an affinity for invasive trophoblasts. A mouse model of FNAIT was developed more than a decade ago and involves immunizing β3-integrin-deficient female mice with wild-type platelets prior to mating with wild-type males (117, 118). Immunization was necessary because β3-integrin-deficient female mice mated to wild-type males do not develop β3-integrin-specific IgG, even after multiple pregnancies. Without considering all conceivable reasons for why this mating fails to elicit FNAIT, perhaps disrupting tolerance to an antigen common to both the placenta and fetus necessitates an additional disturbance in checkpoints that maintain fetomaternal tolerance. Nevertheless, immunizing β3-integrin-deficient female mice with wild-type platelets prior to mating with wild-type males, or passive administration of mouse or human β3-integrin-specific IgG, caused significant fetal loss and thrombocytopenia, hemorrhage, and growth restriction in surviving pups (117, 118). When β3-integrin-deficient female mice were bred to heterozygous males, selective pathology in the β3-integrin^+^ fetuses was observed (120).

In the FNAIT mouse model, β3-integrin-specific IgG led to an accumulation of maternal natural killer (NK) cells in the decidua at E14.5 (120). This time point is well after uterine NK cells have typically declined in normal implantation sites. In E14.5 FNAIT pregnancies, NK cells showed an NKp46^+^CD107^+^ activated phenotype, surrounded maternal spiral arteries that supply the placenta, and appeared to induce apoptosis of β3-integrin^+^ invasive and endovascular trophoblasts (120) (Figure 3b). Optimal maternal placental perfusion requires trophoblast invasion and then successful remodeling of spiral arteries such that they become lined by endovascular trophoblasts. Poor perfusion in these pregnancies thus likely contributed to poor fetal growth. In addition to the placental pathology, fetuses exhibited defects in brain vascular development due to antibody-mediated dysfunction of β3-integrin^+^ vascular endothelial cells (119). Remarkably, the placental pathology, but not fetal thrombocytopenia or intracranial hemorrhage, can be prevented by administering antibodies to deplete NK cells at E11.5 or antibodies to block the activating receptor FcγRIIIa (120). Unfortunately, translating an NK cell depletion strategy to human pregnancy would be risky since uterine NK cells are important for pathogen defense and necessary in early gestation for optimal spiral artery remodeling (121). Nonetheless, the mouse work thus far raises the possibility that immunotherapies that are more selective than IVIg and prednisone could alleviate FNAIT pathology with significantly fewer side effects.

Analyzing clinical studies and placental pathology in cases of human FNAIT illuminates additional interesting facets of fetomaternal tolerance and breaks therein. Why do β3-integrin-specific IgG antibodies cause pathology during pregnancy, but anti-HLA antibodies do not? Curiously, anti-HLA antibodies commonly mediate transfusion reactions to platelets in nonpregnant individuals, yet anti-HLA antibodies are very rarely implicated in FNAIT (113). Since most pregnant women develop anti-HLA antibodies, it remains a conundrum why these do not mediate significant destruction of fetal platelets or endovascular trophoblasts. It may be the case that the broader fetal tissue distribution of HLA dilutes antibody density, thereby reducing effector functionality (113). Or it could be that anti-HLA antibodies are effectively absorbed and destroyed within the placenta, as mentioned above. Frustratingly, at present, we lack an understanding of the factors that shield HLA^+^ endovascular trophoblasts in mice hyperimmunized to paternal strain splenocytes before pregnancy, preventing them from experiencing a fate like that of β3-integrin^+^ trophoblasts in the mouse FNAIT model.

As speculated earlier, antibody glycosylation could alter the effector functionality of certain antipaternal antibodies. This hypothesis was somewhat indirectly evaluated in a recent study attempting to correlate anti-HPA IgG Fc glycans with disease severity (122), which otherwise does not tightly correlate with anti-HPA IgG levels. The Fc region of anti-HPA-1a-specific IgG was found to be modified by glycans that contain significantly less core fucosylation than the glycans attached to total IgG (122). Decreased core fucosylation increased Fc affinity for activating Fc*γ*RIIIa and Fc*γ*RIIIb receptors, enhancing the antibody-dependent phagocytic activity of monocytes. While literature describing placental pathology in human FNAIT pregnancies is relatively sparse, comparison of placentas from FNAIT and control healthy pregnancies showed an increased frequency of chronic inflammatory lesions in FNAIT specimens (115, 123, 124). These included chronic histiocytic intervillositis, a disease characterized by an accumulation of maternal monocytes in the intervillous space, as discussed in the Supplemental Material. Considering these observations and mouse findings, collective evidence suggests the hypothesis that glycan modification of an antipaternal antibody may disrupt fetomaternal tolerance by imparting pathogenic Fc-mediated effector functionality.

## ANTIPHOSPHOLIPID ANTIBODY SYNDROME

Antiphospholipid (aPL) antibodies are a class of autoantibodies present in women prior to pregnancy that elicit complications largely due to their ability to activate complement and cause inflammation in the placenta and uterus. Primary antiphospholipid syndrome (APS) is characterized by serologic evidence of aPL antibodies in the setting of recurrent venous and arterial blood clots and/or adverse pregnancy outcomes (125). Secondary APS refers to cases where these characteristics are seen in a patient with an autoimmune disorder [e.g., 50% of patients with systemic lupus erythematosus (SLE) have APS]. Adverse pregnancy outcomes seen in patients with APS include recurrent miscarriage, fetal growth restriction, preeclampsia, and stillbirth (126). Significantly, aPL antibodies are discovered in up to 25% of pregnancies experiencing fetal growth restriction (127) or recurrent miscarriages (128). Adverse outcomes are often associated with the presence of autoantibodies that recognize β2 glycoprotein I (β2GPI), a phospholipid-binding serum protein (129). β2GPI can associate with trophoblast cell surfaces via binding to the low-density lipoprotein receptor family member ApoER2 (130) or to cardiolipin (131), a phospholipid usually present on inner mitochondrial membranes that becomes exposed on the plasma membrane of apoptotic cells. The biochemistry of β2GPI is complex, and there is a role for β2GPI activity in optimal mouse placental development and function (132). How anti-β2GPI antibodies interact with trophoblast cell surfaces to elicit changes to cell fate and function has been comprehensively reviewed elsewhere (133). Briefly, in vitro experiments show that β2GPI-specific IgG antibodies can inhibit proliferation, hormone production, and invasive properties of trophoblasts (133). In vivo work, however, strongly implicates antibody-mediated effector functions as a key driver of aPL-associated adverse pregnancy outcomes (134–137).

The initial theory explaining the association between aPL antibodies and adverse outcomes posited that thrombus formation in maternal uteroplacental circulation compromised placental function (138). However, this hypothesis did not quite align with the frequent lack of thrombi on pathologic examination (139, 140). Instead, results from more than two decades of research in pregnant mouse models of APS has significantly shifted the paradigm for how aPL antibodies elicit adverse outcomes. Fortuitously, passive administration of human or mouse aPL IgG at various time points before and/or during gestation recapitulated many of the adverse outcomes seen in human APS pregnancies (141). Pregnant mice that received aPL antibodies showed significant increased fetal resorptions, decreased placental weights, and growth restriction in surviving pups when compared with controls (137, 142, 143). Notably, aPL IgG, C3, and neutrophils appeared to accumulate in placentas and deciduas prior to subsequent tissue necrosis (137). Moreover, C3-deficient pregnant mice were protected from the aPL antibody–induced adverse outcomes (137). Therefore, the hypothesis for how aPL antibodies contribute to adverse pregnancy outcomes was revised to involve maternal complement. Reduced pathology in *C4*^−/−^, *C5*^−/−^, and *C5ar1*^−/−^ pregnant mice administered aPL IgG, as well as an absence of pathology in pregnant mice administered F(ab′)_2_ aPL IgG, indicated a requirement for activation of the classical complement pathway by antibody bound to trophoblasts (135). Remarkably, elimination of maternal neutrophils or complement factor B was also protective and associated with significantly reduced C3 accumulation in the uterus (Figure 3c) (135).

Thus, antibody-mediated activation of the classical complement pathway appeared to be required for APS disease initiation. However, without further amplification by the alternative pathway of complement, adverse pregnancy outcomes were largely averted. While it is evident that aPL antibodies can cause damage to the placenta, these findings also highlight a notable degree of trophoblast protection against robust activation of the classical pathway. It will also be intriguing to delve deeper into mechanisms by which trophoblasts appear to avert formation and/or membrane insertion of the membrane attack complex. This phenomenon seems absent not only in this APS mouse model but also in other situations where there is dysregulated activation of the alternative pathway of complement in the placenta (144). Nevertheless, a wealth of evidence indicates that successful pregnancy outcomes necessitate proper placental regulation of complement (83, 144–146), and aPL IgG antibodies are a clear example of maternal antibodies that can override local complement regulatory factors.

Besides fetal loss, APS shows an additional association with preeclampsia (147), a syndrome characterized by imbalances in angiogenic growth factors and poor placental development. Normal placentation requires coordinated local expression of growth factors and their receptors on trophoblasts that invade the decidua and participate in remodeling maternal spiral arteries. Remarkably, innate immune cells, attracted to the placenta due to pathologic complement activation, become sources of tumor necrosis factor alpha (TNF-α) (134) and antiangiogenic factors (145). That neutrophils and monocytes in mouse models of placental complement activation produce excess antiangiogenic factors [e.g., sVEGFR-1 (soluble vascular endothelial growth factor receptor-1)] is somewhat surprising, since these cells often promote angiogenesis in other contexts. Regardless, local production of antiangiogenic factors could impair trophoblast invasion and vascular remodeling, a key mechanistic link for the association between maternal autoantibodies and clinical features of preeclampsia (148).

The mouse model used in pregnancy APS work has inherent limitations, shared with the mouse model for FNAIT. A passively administered bolus of high-dose IgG does not well mimic the sustained levels of aPL IgG antibodies or anti-HPA IgG antibodies in patients. This is particularly problematic for experiments where human IgG antibodies are given to mice, since alloantibody is rapidly cleared and can induce cross-species immunity. These drawbacks necessitate caution when drawing definitive conclusions about requirements for placental pathology in the setting of antiplacental antibodies. It seems probable that along with the presence of high levels of high-affinity, complement-fixing antibodies, the occurrence of a second breakdown in fetomaternal tolerance mechanisms, or the failure of multiple additional mechanisms, is necessary for disease to manifest. For instance, one might hypothesize that antiplacental antibodies become pathogenic when there is an inherited or induced disturbance in trophoblast complement regulatory proteins or cellular damage inflicted via a pathogen. In support of a double-hit hypothesis, clinical evidence shows that women with inherited mutations in complement regulatory factors are at higher risk for pregnancy complications (149).

In total, APS mouse pregnancy studies significantly advanced our understanding of disease pathogenesis and foreshadowed findings from a large prospective study of outcomes in women with SLE. Most important for identifying a high rate of adverse outcomes for African and Hispanic American women, which now requires additional study, the PROMISSE (Predictors of Pregnancy Outcome: Biomarkers in Antiphospholipid Antibody Syndrome and Systemic Lupus Erythematosus) study included assessment of serial blood samples for markers of complement activation (150). In women with APS and/or SLE, elevated serum levels of activated factor B (a marker of alternative pathway activation) and C5b-9 (indicative of terminal complement pathway activation) measured at the end of the first trimester were highly predictive of subsequent adverse outcomes (151). These human findings lend strong support to the hypothesis that excessive activation of complement underlies pregnancy complications in APS.

The current clinical approach to prevent adverse APS pregnancy outcomes is based on the outdated idea of attributing complications to blood clots, prompting prescriptions of anticoagulation medicines. Fortuitously, the partial success achieved in patients receiving heparin seems to be based on its ability to inhibit classical complement pathway activation, rather than its anticoagulant properties (136). Indeed, heparin, but not other anticoagulants devoid of complement inhibition, uniquely offered some protection against fetal loss in pregnancy APS models (136). Based upon the solid mechanistic framework provided by the mouse studies, the IMPACT (Improve Pregnancy in APS with Certolizumab Therapy) clinical trial is currently testing the hypothesis that adding blockade of TNF-α to the current standard of care will prevent imbalances in angiogenic factors and reduce incidence of preeclampsia in APS pregnancies (6).

## THE FUTURE OF B CELL FETOMATERNAL TOLERANCE

Here, in a review focused only on the humoral arm of the maternal adaptive immune system, I first described how B cells and antibodies are impacted by pregnancy physiology and highlighted recent discoveries showing that tolerance is accomplished by antigen-specific pathways. The exceptional conditions where maternal humoral immunity afflicts direct harm to the placenta and fetus were then reviewed. Many topics contained herein are ripe for more discovery, such as the molecular regulation and antigen specificity of IL-10-producing B cells in pregnancy, the role of CD4 T cell help in paternal-specific B cell responses, and the molecular regulation of IgG glycans in pregnancy, to name just a few. One might imagine many creative ways to leverage pregnancy to study questions that lie at the intersection of glycobiology and immunology. And although there have been notable advances in understanding and averting specific adverse pregnancy outcomes, our understanding of the myriad ways in which maternal B cells and antibodies play a role in pregnancy complications also remains at an early stage.

To conclude, I would like to draw attention to the strong need to not only sustain but also increase enthusiasm in fetomaternal tolerance research. The complexity of pregnancy immunology requires recruiting researchers focused on fundamental mechanisms of tolerance, clinicians working directly with pregnant individuals and diagnosing perinatal specimens, and clinician–scientists trained to speak the language of both immune tolerance and maternal–fetal medicine. Robust and redundant immune tolerance mechanisms must be embedded within reproduction since the success of the species requires such. The success of pregnancy in cancer patients who inadvertently received checkpoint blockade during early gestation argues against a singular requirement for these pathways in maternal T cell tolerance of the conceptus (152). Likewise, it is unlikely that a single pathway controls tolerance mediated by maternal B cells and antibodies. It appears then that there remains much to discover about the complex and “well-organized means of rendering maternal and fetal cells during pregnancy innocuous to both individuals” (1, p. 567).

## Supplementary Material

Supplementary Material
