# Supplementary material for: B Cell Responses to the Placenta and Fetus
Source: Annu Rev Pathol. Author manuscript; Available in PMC 2025 Mar 17. (PMC11912550; doi:10.1146/annurev-pathmechdis-111523-023459)
Supplement: Supplementary Material [file NIHMS2064364-supplement-Supplementary_Material.docx]

**Supplemental Material**

***Enigmatic placental pathologies indicative of an antibody-mediated rejection phenomenon?***

Here, attention is drawn to three enigmatic placental conditions: Villitis of Unknown Etiology (VUE), Chronic Histiocytic Intervillositis (CHIV), and Massive Perivillous Fibrin Deposition (MPVFD). All show abnormal infiltration of maternal monocytes, and/or T cells in or around placental villi, variable amounts fibrin accumulation, and tissue necrosis (1). While a complete discussion of clinical and diagnostic features is outside the scope here, I would like to highlight features suggestive of anti-placental antibody involvement in pathogenesis and emphasize a need for research aimed at elucidating etiology. It is important to note that diagnoses of VUE and CHIV are based on the exclusion of other conditions, given their striking histologic resemblance to various infections. Recent research significantly challenges this notion, drawing similarity between immune milieu of affected placentas and that of rejected organ grafts (2-5). Unfortunately, at present, all three can only be detected and diagnosed upon examination of the placenta. High-grade cases of VUE, CHIV, and MPVFD are associated with severe fetal growth restriction and stillbirth, and there is a significant risk of recurrence with increasing severity in subsequent pregnancies with the same partner (6). Thus, understanding their pathogenesis is an important priority.

It is reasonable to hypothesize that VUE, CHIV, and MPVFD involve maternal conceptus-specific B and T cells. Focusing on similarities between all three, histologic evidence of complement activation products (e.g., C4d, C5b-9) deposited along a damaged syncytiotrophoblast layer has been reported (4; 5; 7; 8). Analysis of small numbers of cases shows concurrence of high maternal serum levels of fetal HLA-specific IgG with complement fixing capacity and histologic evidence of complement activation product deposition along placental villi with HLA^+^ syncytiotrophoblasts (4), which can also show aberrant expression of the leukocyte adhesion molecule ICAM-1 (9). Syncytiotrophoblasts lack expression of HLA and ICAM-1 adhesion molecule under normal conditions. Remarkably, some cases meet all histologic requirements for diagnosis of “antibody-mediated rejection” as delineated by the gold-standard Banff classification for allograft pathology (4).

By leveraging our existing knowledge of fetomaternal tolerance mechanisms and conditions where tolerance fails, a model for pathogenesis of these mysterious placental diseases begins to take shape. First, antigenic quantities of fetal blood cells enter maternal circulation (“fetomaternal hemorrhage”). This occurs via disruption in the syncytiotrophoblast layer and underlying fetal endothelium. While external trauma appears to promote fetomaternal hemorrhage, tiny hemorrhages occur late in normal gestation and could be a consequence of trophoblast aging and accumulation of deleterious mutations or turbulent intervillous blood flow. Regardless, direct allorecognition of paternal HLA might then activate maternal CD4 and CD8 T cells, with HLA-specific B cells receiving help from cognate CD4 T cells. Note that, based upon our current knowledge, this degree of alloreactivity, where the mother develops anti-paternal antibodies, occurs in most pregnancies. What then distinguishes rare cases that progress to disease? Using CHIV as an example suggests the hypothesis that additional failures in fetomaternal tolerance gives rise to these diseases. CHIV is defined by infiltration of the intervillous space by maternal CD68^+^ monocytes, some of which appear to adhere to syncytiotrophoblast covered in complement cleavage products. In healthy pregnancies, perhaps trophoblast complement regulatory proteins and/or maternal anti-placental antibodies with anti-inflammatory Fc glycans, can suppress inflammation. Perhaps, in the very rare cases of CHIV, anti-placental IgG with complement fixing capacity and pro-inflammatory Fc glycans bind with high affinity to the syncytiotrophoblast layer and activate the classical complement pathway which goes unmitigated due to inherited or acquired defects in complement regulation. Then, anaphylatoxins recruit maternal myeloid cells which impair trophoblast function via inflammatory cytokines and antibody-dependent cellular cytotoxicity. At present this is purely speculative, however it is possible that future immunologic, genetic, and biochemical studies can test this model and illuminate true etiology.

**Supplemental references**

1. Roberts DJP, C. 2021. *Atlas of Placental Pathology* American Registry of Pathology

2. Enninga EAL, Raber P, Quinton RA, Ruano R, Ikumi N, et al. 2020. Maternal T Cells in the Human Placental Villi Support an Allograft Response during Noninfectious Villitis. *J Immunol* 204:2931-9

3. Romero R, Whitten A, Korzeniewski SJ, Than NG, Chaemsaithong P, et al. 2013. Maternal floor infarction/massive perivillous fibrin deposition: a manifestation of maternal antifetal rejection? *Am J Reprod Immunol* 70:285-98

4. Benachi A, Rabant M, Martinovic J, Bouchghoul H, Vivanti AJ, et al. 2021. Chronic histiocytic intervillositis: manifestation of placental alloantibody-mediated rejection. *Am J Obstet Gynecol* 225:662 e1- e11

5. Bendon RW, Coventry S, Thompson M, Rudzinski ER, Williams EM, Oron AP. 2015. Significance of C4d Immunostaining in Placental Chronic Intervillositis. *Pediatr Dev Pathol* 18:362-8

6. Cornish EF, McDonnell T, Williams DJ. 2022. Chronic Inflammatory Placental Disorders Associated With Recurrent Adverse Pregnancy Outcome. *Front Immunol* 13:825075

7. Kim EN, Lee JY, Shim JY, Hwang D, Kim KC, et al. 2019. Clinicopathological characteristics of miscarriages featuring placental massive perivillous fibrin deposition. *Placenta* 86:45-51

8. Rudzinski E, Gilroy M, Newbill C, Morgan T. 2013. Positive C4d Immunostaining of Placental Villous Syncytiotrophoblasts Supports Host-Versus-Graft Rejection in Villitis of Unknown Etiology. *Pediatric and Developmental Pathology* 16:7-13

9. Juliano PB, Blotta MH, Altemani AM. 2006. ICAM-1 is overexpressed by villous trophoblasts in placentitis. *Placenta* 27:750-7
